# Supplementary material for: Correlation between Fatty Acid Profile and Anti-Inflammatory Activity in Common Australian Seafood by-Products
Source: Mar Drugs. 2019 Mar 6;17(3):155. doi: 10.3390/md17030155 (PMC6471488; doi:10.3390/md17030155)
Supplement: Supplementary file 1 [file marinedrugs-17-00155-s001.pdf]

marine drugs

MDPI

Supplementary Materials

Correlation between Fatty Acid Profile and Anti-Inflammatory Activity in Common Australian Seafood by-Products

Tarek B. Ahmad [1,2,†], David Rudd [1,3], Michael Kotiw [2], Lei Liu [4] and Kirsten Benkendorff [1,†,*]

1 Marine Ecology Research Centre, Southern Cross University, Lismore, 2480, Australia; Tarek.Ahmad@usq.edu.au (T.B.A.); david.rudd@monash.edu (D.R.)
2 Division of Research & Innovation, University of Southern Queensland, Toowoomba, 4350, Australia; Michael.Kotiw@usq.edu.au
3 Monash Institute of Pharmaceutical Sciences, Monash University, Parkville, 3052, Australia
4 Southern Cross Plant Science, Southern Cross University, Lismore, 2480, Australia; Ben.liu@scu.edu.au
* Correspondence: kirsten.benkendorff@scu.edu.au; Tel.: +61-2-6620-3755
† These authors contributed equally to the manuscript.

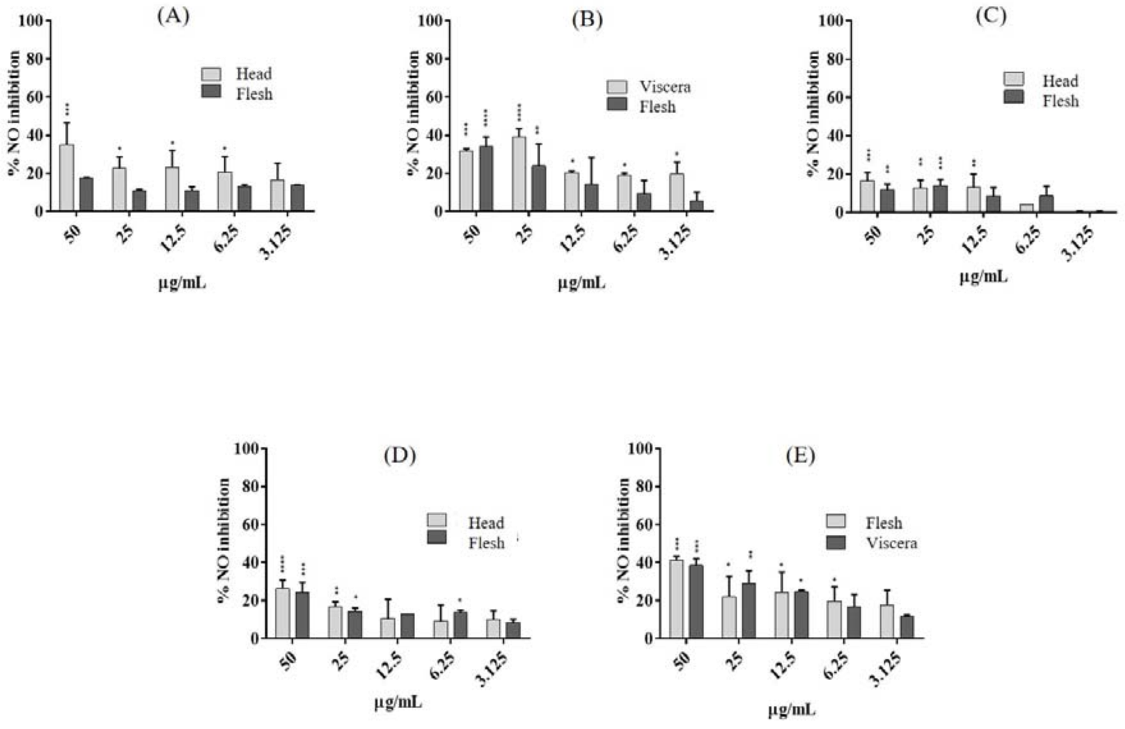

Figure S1. The NO inhibitory activity of lipid extracts from different seafood organisms; (**A**) *Penaeus plebejus* (Australian school prawn), body flesh and head, including viscera; (**B**) *Sardinops sagax* (Australian sardine) flesh and viscera, including heads; (**C**) *Salmo salar* (Atlantic salmon) flesh and heads; (**D**) *Sepioteuthis australis;* (**E**) *Octopus tetricus* *$p < 0.05$, **$p < 0.01$, ***$p < 0.001$, ****$p < 0.0001$ versus the LPS + Solvent control.

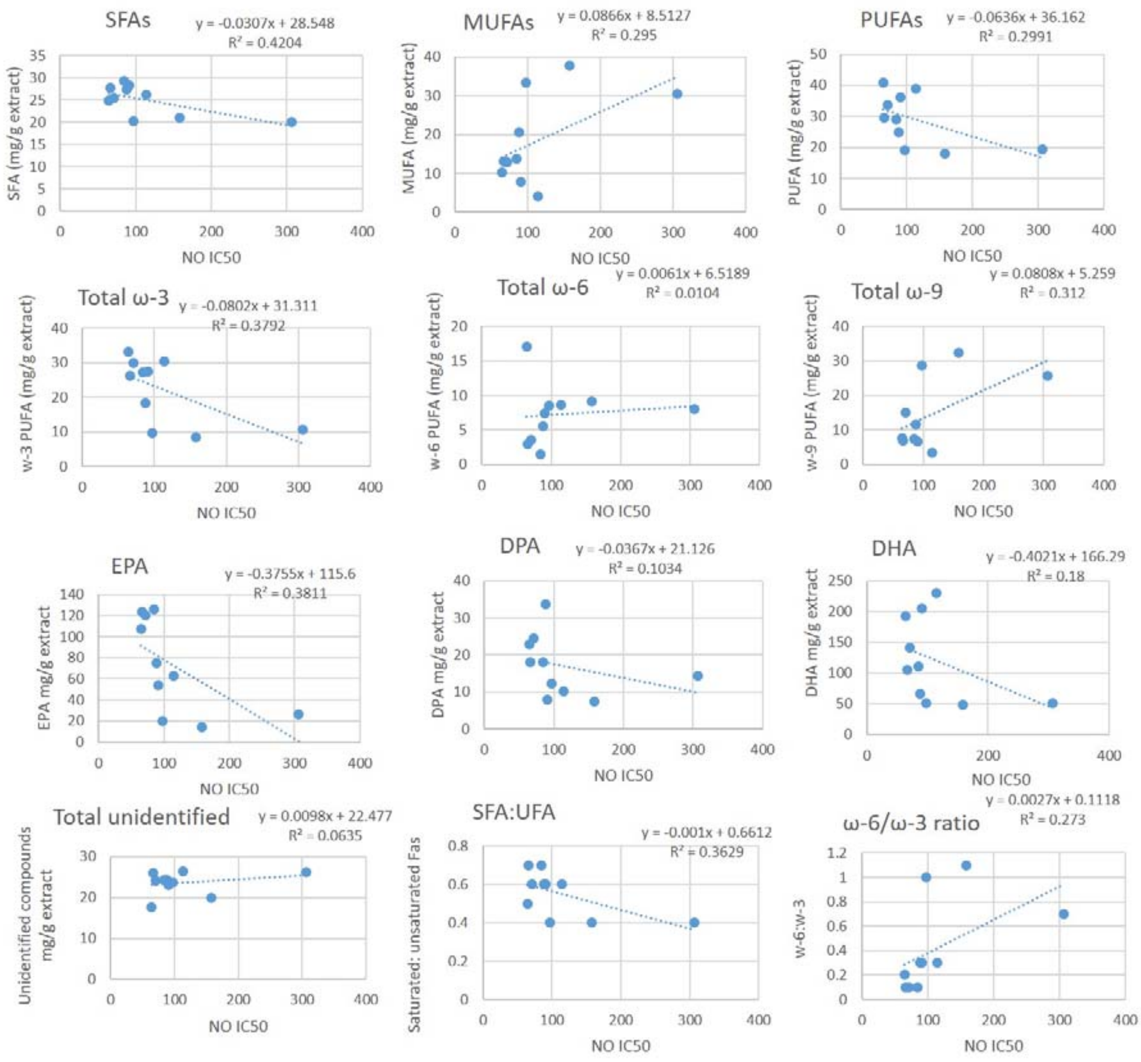

Figure S2. Correlations between NO inhibitory activity (IC_{50}) of lipid extracts and the amount of certain fatty acid classes or ratios in different seafood organisms.

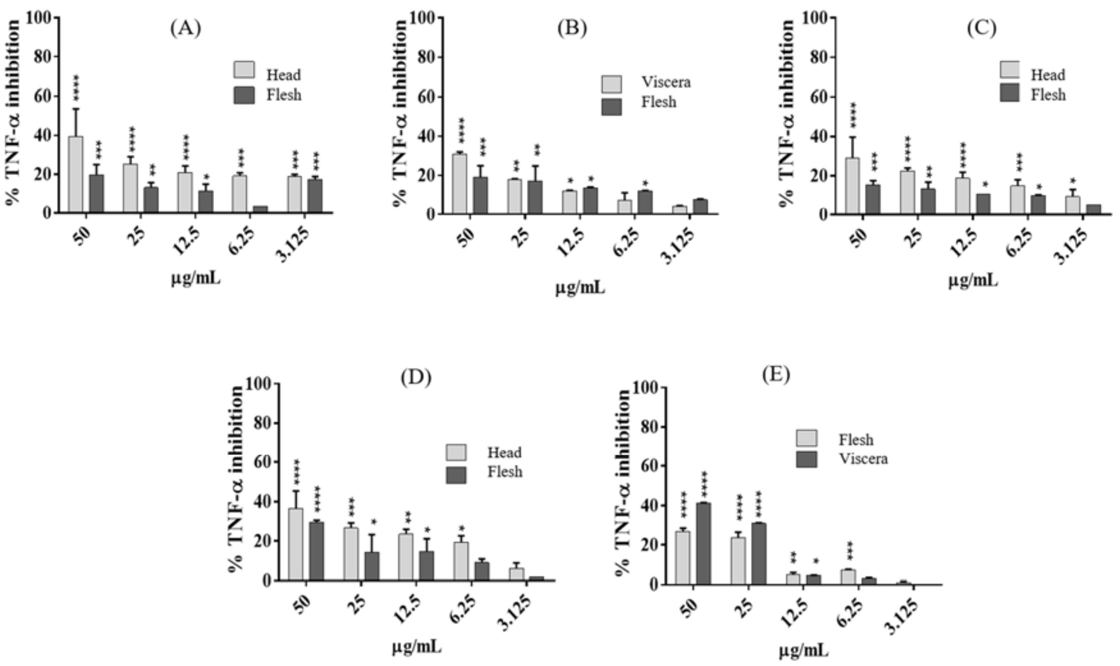

Figure S3. The TNFα inhibitory activity of lipid extracts from different seafood organisms; (**A**) *Penaeus plebejus* (Australian school prawn), body flesh and head, including viscera; (**B**) *Sardinops sagax* (Australian sardine) flesh and viscera, including heads; (**C**) *Salmo salar* (Atlantic salmon) flesh and heads; (**D**) *Sepioteuthis australis;* (**E**) *Octopus tetricus* *p < 0.05, **p < 0.01, ***p < 0.001, ****p < 0.0001 versus the LPS + Solvent control.

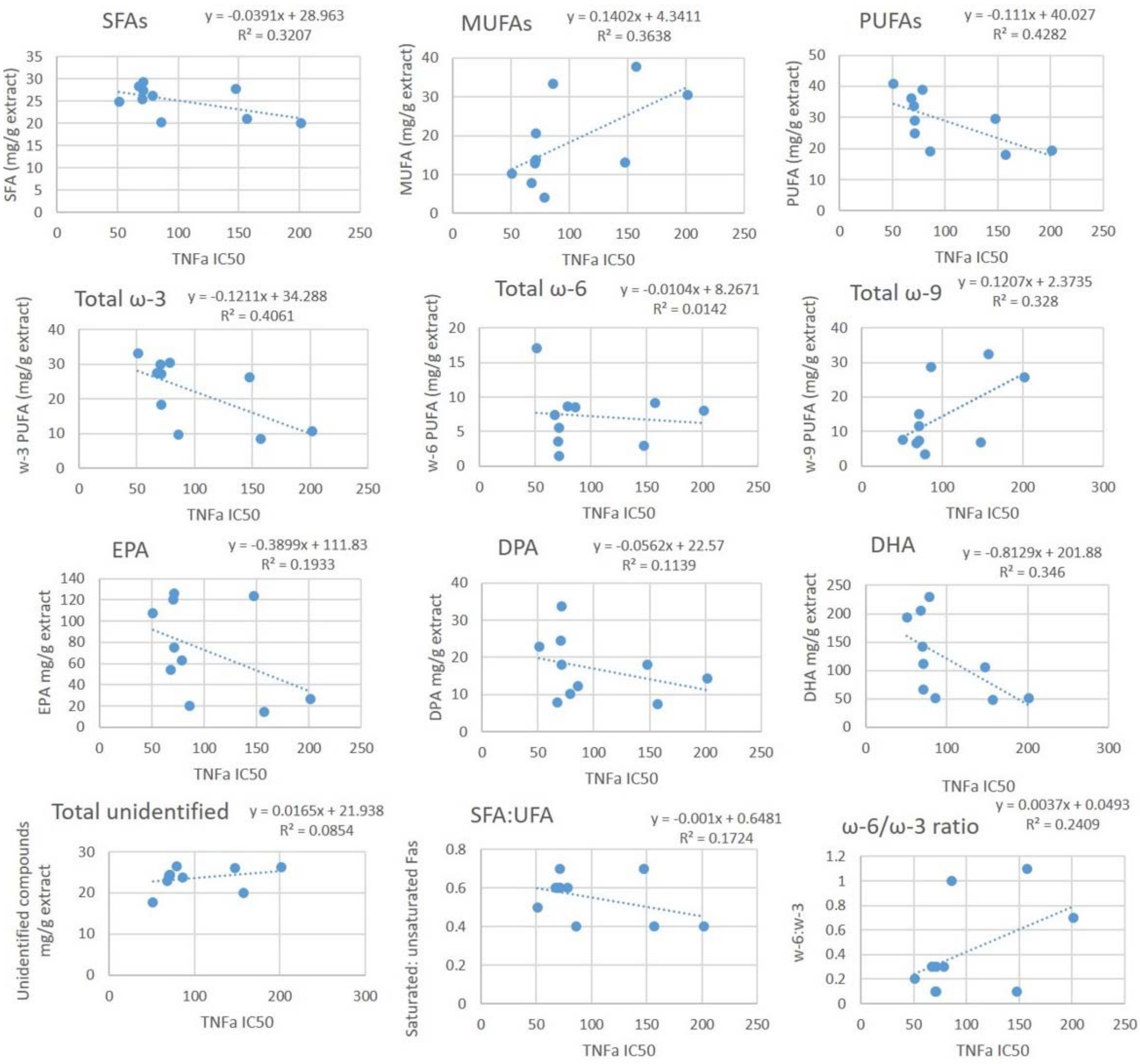

Figure S4. Correlations between TNFα inhibitory activity (IC_{50}) of lipid extracts and the amount of certain fatty acid classes or ratios in different seafood organisms.
